# Pre-Cueing Effects: Attention or Mental Imagery?

**DOI:** 10.3389/fpsyg.2017.00222

**Published:** 2017-03-06

**Authors:** Peter Fazekas, Bence Nanay

**Affiliations:** ^1^Philosophy and Cognitive Neuroscience Research Unit, Aarhus UniversityAarhus, Denmark; ^2^Centre for Philosophical Psychology, University of AntwerpAntwerp, Belgium; ^3^Department of Philosophy, University of AntwerpAntwerp, Belgium; ^4^Peterhouse, University of CambridgeCambridge, England

**Keywords:** attentional modulation, baseline activity, filtering, mental imagery, cognitive penetration

## Attention and cognitive penetrability

Much has been written recently about cognitive penetration. If there are perceptual computations that are directly influenced by the information content of certain cognitive states such that the changes in the output of these computations can be accounted for in terms of the content of the penetrating cognitive states, we can talk about the cognitive penetration of perceptual processing.^1^

When considering the possible mechanisms that could mediate cognitive penetration, attention, traditionally, is quickly sidelined as a phenomenon that is trivially unable to exert the right kind of effect on perception. Even if the allocation of goal-directed (top-down, endogenous) attention is driven by the content of certain cognitive states (i.e., goal representations), it does not have a direct influence on perceptual processing itself. For, according to the traditional characterization, attention acts as a filter, a gatekeeper (Broadbent, 1958), or a spotlight (Posner, 1980) that selects and enhances certain signals (corresponding to attended stimuli) while attenuating or filtering out competing signals “prior to the operation of early vision” (Pylyshyn, 1999: p. 344).

This traditional understanding has recently been questioned by empirical findings demonstrating that attention is not a passive gatekeeper mechanism acting before the start of perceptual processing, but rather an active modulator of perceptual computations that is able to exert many different effects at many different levels of the perceptual hierarchy (see e.g., Reynolds and Chelazzi, 2004; Nanay, 2010b; Noudoost et al., 2010; Carrasco, 2011, 2014; Lupyan, 2015; Wu, in press).

However, despite this transition from seeing attention as passive gatekeeper to seeing it as an active modulator, opponents still argue against attention-mediated cognitive penetration on the basis of the filter-like nature of attention. As Firestone and Scholl have recently put it, attending is “importantly analogous to seeing through a tinted lens —merely increasing sensitivity to certain features rather than others” (Firestone and Scholl, 2017: p. 23, but see also Lupyan, in press).

## Pre-cueing and attentional modulation

Thinking about attention as a filter, even in the light of recent experimental data and conceptual shift is supported by some of the empirical findings.

At the behavioral level, attention increases processing efficiency: The allocation of attention enhances detection rates, speeds reaction times, increases accuracy (Posner, 1980; Posner et al., 1980; Castiello and Umiltà, 1990; Carrasco, 2011). Neural level studies suggest that attention achieves all these by enhancing the neural signals encoding the stimulus-features in question, i.e., by modulating the behavior of sensory neurons in various ways, including amplifying neural responses (Carrasco, 2011), sharpening response functions (Martinez-Trujillo and Treue, 2004; Maunsell and Treue, 2006), and remapping receptive fields (Anton-Erxleben and Carrasco, 2013). Most importantly from our present perspective, attention amplifies neural responses via multiplicative effects like evoking response gain or contrast gain, and also via additive effects like increasing baseline activity (Buracas and Boynton, 2007; Carrasco, 2011; Cutrone et al., 2014).

Pre-stimulus cues increase related baseline activity well before the occurrence of the stimulus (Chawla et al., 1999; Reynolds et al., 2000). This enhanced baseline or spontaneous activity correlates with increased behavioral performance such that subjects with large modulation of baseline activity perform better once the stimulus is presented (Giesbrecht et al., 2006). That is, with a pre-stimulus boost of the spontaneous activity of neurons tuned toward a target the sensitivity of these neurons is increased, and therefore stimulus processing is enhanced.

One way of describing these findings, and one that is standard in the literature, is that this is an attentional effect. When attention is turned toward a specific spatial region or a particular feature value, the activity of cortical neurons selectively responding to the specific spatial region or particular feature value increases. Pre-cueing studies show that this can even be true without the presence of any stimuli in the specific region or with the particular feature. In those cases, top-down attentional modulation increases the activity of those neurons which are sensitive to the spatial position or feature value indicated by the endogenous pre-cue. Since this process is driven by cognitive contents, this provides a nice demonstration of the cognitive penetration of perception by attention.

However, if we construe these studies this way, then the concept of attention at play here will be attention that does act very much like a filter—not as a mere gatekeeper simply letting through some stimuli while blocking others, but as a more advanced filter that is able to modulate certain features of the light passing through it. Also note that attention exerts this effect *before* stimulus presentation, i.e., well before the start of stimulus processing. That is, in these cases it seems that the opponent of attention mediated cognitive penetration could run a very simple objection: Attention does not seem to affect perceptual processing itself, not at least in a direct way; it only increases the sensitivity of processing units, readying them for the stimuli to come. In short, everything the pre-cueing studies show us about attention would be consistent with a Pylyshyn-esque picture of cognitive impenetrability: There are top-down attentional effects at the entry-level of perceptual processing, but not afterwards.

## Pre-cueing and mental imagery

As we have seen, the claim that pre-cueing studies show that perception is cognitively penetrated by means of attentional mechanisms is problematic. Nevertheless, we do want to argue that pre-cueing studies show that perception is cognitively penetrated—not via the mediation of attention, but via mental imagery. In what follows we will argue that cue-induced mental imagery provides a channel through which cognitive states can exert such effects on perception that fulfill the requirements of cognitive penetration.

The concept of mental imagery has been controversial, but we want to use a fairly non-demanding characterization, going back to Kosslyn, Behrmann, and Jeannerod: “Visual mental imagery is “seeing” in the absence of the appropriate immediate sensory input, auditory mental imagery is “hearing” in the absence of the immediate sensory input, and so on. Imagery is distinct from perception, which is the registration of physically present stimuli.” (Kosslyn et al., 1995, p. 1335). This is the sense in which contemporary psychology and neuroscience (but not philosophy) talks about mental imagery. Just one example from a recent review article: “We use the term “mental imagery” to refer to representations […] of sensory information without a direct external stimulus” (Pearson et al., 2015). We can summarize this concept as “perceptual processing that is not triggered by corresponding sensory stimulation in the relevant sense-modality” (Nanay, 2016).

Note that mental imagery, understood this way does not have to be voluntary, it is often involuntary (in flash-backs or in the case of earworms). It does not have to be conscious either (if sensory stimulation-driven perceptual processing can be unconscious, then so can perceptual processing that is not triggered by corresponding sensory stimulation). And while it is typically driven by top-down information, it can also be triggered laterally (by information in another sense modality) or in a bottom-up manner (as in the case of the blind spot, where the information is provided by the regions of the retina around the blind spot). It is also important to note that by “perceptual processing” what is meant in these definitions is “early cortical processing”—in the case of the visual sense modality, for example, we have early cortical activation in the primary visual cortex that does not correspond to the retinal activation.

Pre-cueing studies could be interpreted in this theoretical framework as instances of mental imagery: Pre-cueing induces early perceptual processing (as early as V1) that is not triggered by corresponding sensory stimulation in the relevant sense modality (that is, by corresponding retinal activation). In other words, given the definition of mental imagery above, pre-cueing induces mental imagery of the pre-cued feature. This is true of pre-cueing for a number of features, such as shape, color, and motion (see Shibata et al., 2008 for a good summary, see also Zhuang and Papathomas, 2011).

Mental imagery interacts with the perceptual processing of stimuli at all relevant stages of the perceptual hierarchy, starting with the earliest one. Early cortical processing of presented stimulus during mental imagery leads to a mixed imagery/perception state, where the activation of the V1, for example, is partially determined by the visual stimulus and partly by mental imagery. This is the clearest in the studies of illusory contours, where the early perceptual processing of illusory contours (in V1 and V2) is a mixture of amodal completion (which comes out as mental imagery according to our definition) and stimulus-driven processes (Kovács et al., 1995; Sugita, 1999; Bakin et al., 2000; Lee and Nguyen, 2001; Komatsu, 2006; Hedgé et al., 2008; Lommertzen et al., 2009; Vrins et al., 2009; Nanay, 2010a; Smith and Muckli, 2010; Bushnell et al., 2011; Shibata et al., 2011; Lee et al., 2012; Pan et al., 2012; Ban et al., 2013; Emmanouil and Ro, 2014; Hazenberg et al., 2014; Scherzer and Ekroll, 2015).

Some instances of amodal completion may be fully bottom-up driven, like the completion of simple shapes purely on the basis of Gestalt forms (that can go against our best judgments). But some other times, amodal completion is driven in a top-down manner, for example, in the case of seeing the cat behind the picket fence. Depending on what cats I encountered before, the way I complete this figure would be very different. The same goes for the amodal completion of letters and words.

One experimentally controlled study of top-down driven amodal completion (that is, mental imagery according to the definition above) and the way it interacts with perception comes from studies of how we perceive two-tone pictures before and after information is given about what the picture is of Teufel et al. (2015). Here, the mental imagery we use to complete the illusory contours very much depends on top-down information and this influences very early (V1) perceptual processing.

Because of the multiple and very early interactions between the perceptual processing of stimuli and mental imagery, mental imagery influences the way stimuli will get processed throughout perception (as opposed to exerting modulatory effects only at the input of early perceptual processing) thereby avoiding Pylyshyn-esque lines of objection. And given that most instances of mental imagery depend on content-driven top-down influences (Macpherson, 2012), this means that mental imagery can modulate perceptual computations in a direct, top-down, content sensitive manner.

This is our argument for the claim that pre-cueing studies show that perception is cognitively penetrated via mental imagery. It is important to be clear about the relation between attention and mental imagery here. We do not want to question the role of attention in pre-cueing studies. After all, it is attention that is being pre-cued. The pre-cue draws attention to certain features, which via top-down connections induces mental imagery for the pre-cued properties, which, then, after stimulus-presentation, interacts with and influences the online computations that process stimulus features. That is, what mediates the cognitive penetration of perceptual processing is not pre-cued attention, but cue-induced mental imagery.

## Author contributions

All authors listed, have made substantial, direct and intellectual contribution to the work, and approved it for publication.

## Funding

The study was supported by the Danish Council for Independent Research & FP7 Marie Curie Actions - COFUND DFF-Mobilex Mobility Grant 1321-00165, and the FWO Postdoctoral Fellowship 1.2.B39.14N (PF).

### Conflict of interest statement

The authors declare that the research was conducted in the absence of any commercial or financial relationships that could be construed as a potential conflict of interest.

## Footnotes

^1^See, for example, the much-cited passage from Zenon Phylyshyn: “if a system is cognitively penetrable then the function it computes is sensitive, in a semantically coherent way, to the organism's goals and beliefs, that is, it can be altered in a way that bears some logical relation to what the person knows” (Pylyshyn, 1999: p. 343). Pylyshyn was interested in the cognitive penetrability of early visual processing, whereas in contemporary discussion the emphasis has been shifted to perceptual processing underlying conscious experiences (Macpherson, 2012, see also Teufel and Nanay, 2017 on the distinction). We will concentrate on the former question here. Recently, Raftopoulos (2009, 2014) has offered a definition of early vision in terms of perceptual processing occurring within 120 ms after stimulus presentation (see also Raftopoulos and Zeimbekis, 2015). Our focus on pre-cueing effects ensures that our claims are applicable even to this characterization of early visual processing.
